# Quasispecies Analysis of JC Virus DNA Present in Urine of Healthy Subjects

**DOI:** 10.1371/journal.pone.0070950

**Published:** 2013-08-15

**Authors:** Tom Van Loy, Kim Thys, Luc Tritsmans, Lieven J. Stuyver

**Affiliations:** 1 Janssen Diagnostics, Beerse, Belgium; 2 Janssen Infectious Diseases – Community of Research Excellence & Advanced Technology (C.R.E.A.Te), Beerse, Belgium; 3 Janssen Neuroscience Development, Beerse, Belgium; Saint Louis University, United States of America

## Abstract

JC virus is a human polyomavirus that infects the majority of people without apparent symptoms in healthy subjects and it is the causative agent of progressive multifocal leucoencephalopathy (PML), a disorder following lytic infection of oligodendrocytes that mainly manifests itself under immunosuppressive conditions. A hallmark for JC virus isolated from PML-brain is the presence of rearrangements in the non-coding control region (NCCR) interspersed between the early and late genes on the viral genome. Such rearrangements are believed to originate from the archetype JC virus which is shed in urine by healthy subjects and PML patients. We applied next generation sequencing to explore the non-coding control region variability in urine of healthy subjects in search for JC virus quasispecies and rearrangements reminiscent of PML. For 61 viral shedders (out of a total of 254 healthy subjects) non-coding control region DNA and VP1 (major capsid protein) coding sequences were initially obtained by Sanger sequencing. Deletions between 1 and 28 nucleotides long appeared in ∼24.5% of the NCCR sequences while insertions were only detected in ∼3.3% of the samples. 454 pyrosequencing was applied on a subset of 54 urine samples demonstrating the existence of JC virus quasispecies in four subjects (∼7.4%). Hence, our results indicate that JC virus DNA in urine is not always restricted to one unique virus variant, but can be a mixture of naturally occurring variants (quasispecies) reflecting the susceptibility of the non-coding control region for genomic rearrangements in healthy individuals. Our findings pave the way to explore the presence of viral quasispecies and the altered viral tropism that might go along with it as a potential risk factor for opportunistic secondary infections such as PML.

## Introduction

Human polyoma viruses (HPyVs) are non-enveloped DNA viruses with a ∼5 kilobase pair circular, double-stranded DNA genome. Several members of this virus family are associated with human pathologies. JC virus (JCV) infection can result in a severe clinical outcome *i.e.* progressive multifocal leucoencephalopathy (PML), an often fatal neurological disorder resulting from a demyelination process after lytic infection of oligodendrocytes [1], [2]. PML occurs predominantly in patients suffering from immune deficiencies (*e.g.* HIV-patients) or patients undergoing immuno-modulatory therapies. For instance, PML-cases have been reported for patients treated with immuno-modulatory monoclonal antibodies such as natalizumab and rituximab [3], [4]. BK virus (BKV), the polyomavirus most closely related to JCV, has been shown to be involved in nephropathy after kidney transplant and may also be an opportunistic pathogen of the central nervous system [5], [6], [7]. Also JCV has been associated with nephropathy [8]. More recently, other human polyoma viruses such as Merkel cell polyomavirus (MCV) and TSPyV have also raised interest as oncogenic viruses [9], [10], [11].

The genome of JC virus encodes both structural and regulatory proteins [12]. Small t-antigen (stAg) and large T-antigen (LTAg) are early expressed regulatory proteins resulting from alternative splicing of the same primary transcript and play a key role in viral replication [13], [14]. Late genes encode the viral capsid proteins VP1, VP2, and VP3, as well as agnoprotein, another regulatory protein. Expression of early and late genes is regulated by a bi-directional non-coding control region (NCCR) which is positioned on the genome between the coding parts of the early and late genes [12]. This control region carries the viral origin of replication (*ori*) immediately followed by DNA sequence motifs recognized by host transcription factors. As such the NCCR is a key determinant in regulating viral replication and early and late transcription events in host cells permissive for JCV infection, as has been experimentally shown in several *in vitro* cell studies [15], [16], [17].

Within an infected host at least two main JC virus variants can exist that differ predominantly in the organization of the NCCR. In the current view, following infection a non-pathogenic form of JC virus can persist in a variety of cell types including, but not limited to, kidney epithelial cells, tonsils and B cell precursors in bone marrow [18]. Primary infection in general occurs without apparent symptoms. Based on the prevalence of anti JCV-antibodies directed against the major capsid protein VP1, it has been estimated that the vast majority of humans have experienced JC virus infection, often already during childhood [18]. A subpopulation of infected people also shed JCV DNA in their urine (viruria). The naturally occurring JCV variant found in urine of both healthy individuals as well as PML-patients is known as the archetype JC virus and is characterized by a typical, well-conserved architecture of the non-coding control region *i.e.* it is build up of a defined sequence of DNA motifs referred to as domain a to f [19], [20]. JC virus isolated from brain and cerebrospinal fluid (CSF) from patients diagnosed with PML typically carries multiple genomic rearrangements in the NCCR [17], [21] that are believed to have evolved from the archetype virus by deletions and duplications [19] and that can be very hyper variable between individual PML-cases [21].

Since archetype JC virus has not been associated with PML NCCR rearrangements (*i.e.* insertions and deletions) might be a driving force for viral replication and gene transcription in glial cell types ultimately triggering the lytic phase as suggested by previous reports [17], [22]. Although single nucleotide differences between NCCR sequences are not uncommon, their functional impact remains largely unknown [17], [21], [23]. Hence, since the accumulation of DNA rearrangements in the NCCR might alter JCV tropism by changing DNA binding sites for cellular transcription factors in cells permissive for infection [15], [24] investigating the naturally occurring variability in the non-coding control region of JC virus and the presence of potential quasispecies seems of particular interest. In addition, upon potential dissemination of the virus within the host, these rearrangements might support viral replication in cell types other than the primary sites of infection.

As a first step in this thinking, we set up a detailed analysis of the presence of genomic rearrangements and nucleotide variability in the non-coding control region of JC virus DNA as it is isolated from urine of healthy subjects. As a gold standard, Sanger sequencing was used to determine the NCCR sequence and the VP1 coding sequence for the examined samples. Next, 454 amplicon sequencing was applied on a subset of 54 samples to study in detail the presence of potential JCV quasispecies. Our data provide evidence for the existence of JC virus quasispecies within naturally infected hosts and shed light on the degree of variability present in the JC virus NCCR DNA.

## Materials and Methods

### Ethics statement

The Ethics Committee [“Commissie voor Medische Ethiek - ZiekenhuisNetwerk Antwerpen (ZNA) and the Ethics committee University Hospital Antwerp] approved the Protocols, and Informed consents, which were signed by all subjects.

### Healthy subject samples

A total of 254 healthy subjects (HSs) were selected in Belgium for this study: 135 women and 119 men with an age ranging from 19 up to 66 years (median 42 y, average 42.2 y, 25% percentile 35 y, 75% percentile 50 y). 36 of these HSs donated one or more consecutive urine samples over a time interval of one year. Urine samples were collected and stored at −80°C until further processing.

### JC virus viral load assay

DNA was extracted from 200 µl or 1 ml urine aliquots using the NucliSENS® easyMAG® reagents and platform (Biomérieux). DNA was eluted in 25 µl final volume. The presence of JC virus DNA was determined by quantitative Polymerase Chain Reaction (qPCR) utilizing a previously described primer set (*i.e.* JCT-1, JCT-2 ) and a FAM/TAMRA labeled internal probe (JCT-1.1) designed to amplify a JC virus large T (LTAg) gene fragment [25]. To quantify the viral load the targeted LTAg gene fragment was subcloned into a pMA backbone (Life Technologies). A 10-fold serial dilution of linearized plasmid DNA was prepared covering a dynamic range of 10 to 10^8^ calculated copy numbers per 5 µl. Another plasmid carrying the homologuous BK virus LTAg gene fragment was prepared similarly and included as negative control plasmid. For each sample a 15 µl pre-PCR mixture was prepared containing: 10 µl LightCycler® Probe master (2×) (Roche), 0.06 µl primer JCT-1 (100 µM), 0.06 µl primer JCT-2 (100 µM), 0.04 µl probe JCT-1.1 (100 µM) and 4.84 µl PCR grade water. 5 µl of DNA extracted from urine, plasmid DNA or PCR grade water (*i.e.* no template control) was added. Samples were run in duplicate on the BioRad CFX 96 thermocycler with following cycling conditions: 95°C for 5 min, followed by 40 cycles of 95°C for 10 s, 60°C for 10 s and 72°C for 10 s. qPCR data were analyzed with the BioRad CFX Manager^TM^ software v2.1. and the JC virus VL calculated and expressed as log copies per ml urine.

### Sanger sequencing of JC virus non-coding control region and VP1 coding sequence

Viral DNA was extracted from urine as described above and used as template for outer PCR using Phusion high fidelity master mix (2×) (New England Biolabs) and template specific primers: [5′ gattcctccctattcagcactttg 3′ (Fwd primer) and 5′ tccactccaggttttactaa 3′ (Rev primer); JCV NCCR] and [5′ cctcaatggatgttgccttt 3′ (Fwd primer) and 5′ aaaaccaaagacccctc 3′ (Rev primer); JCV VP1 coding sequence]. Cycling conditions for PCR were: 98°C for 30 seconds followed by 40 cycles of 98°C for 10 sec, 60°C for 20 sec and 72°C for 20 sec and a final step at 72°C for 5 min. Generated PCR products were subsequently used as template for sequencing PCR using BigDye termination sequencing reagents (Applied Biosystems) and sequencing primers: [5′ ctattcagcactttgtccattttagc 3′ (Fwd primer NCCR) and 5′ ggttttactaactttcacagaagcct 3′ (Rev primer NCCR] or [5′ctttacttttagggttgtacgggac 3′ (Fwd primer1 VP1); 5′ tgaggatctaacctgtggaa 3′ (Fwd primer2 VP1); 5′ctcccccaaaataactgcaact 3′ (Rev primer1 VP1) and 5′ tcctctccactgctggga 3′ (Rev primer2 VP1). Sequencing PCR was run as follows: 96°C for 1 min followed by 35 cycles of 96°C for 10 sec, 50°C for 5 sec and 60°C for 4 min. Samples were purified [DyeEx 2.0 Spin kit (Qiagen)] and run on the 3730×l DNA Analyzer (Applied Biosystems). DNA sequences were analyzed with the SeqScape v2.5 software. Non-coding control region DNA sequences harboring rearrangements were submitted to EMBL/GenBank (accession numbers HF955438 to HF955450).

### Phylogenetic analysis of HS samples

Full length VP1 coding sequences (1065 bp) obtained for HS samples (n = 61) were used for phylogenetic analysis. All sequences were first compared to VP1 coding sequences retrieved from JC virus genotype reference strains [26] in a multiple sequence alignment using clustalW2 (http://www.ebi.ac.uk/Tools/msa/clustalw2/). Data were further processed with the MEGA 5.05 software to generate a phylogenetic tree using Neighbour Joining method. The VP1 coding sequence of JCV genotype 6 was used to root the tree. All different VP1 coding sequences have been submitted to EMBL/GenBank and have accession numbers HF955451 to HF955498.

### 454 amplicon sequencing on GS Junior platform

#### Control plasmids included in the study

An archetype JC virus non-coding control region DNA sequence (CY isolate, NCBI acc.nr. AB038249) was subcloned into the pMA backbone and used as template for PCR amplification. A similar plasmid, but in which a predefined 66 base pairs (bps) deletion corresponding to domain d of the JCV NCCR was introduced, was also generated. XL gold ultracompetent *E. coli* cells were transformed with both plasmids according to standard procedures and single isolated colonies were cultured overnight at 37°C in 3 ml LB-medium under selection of 100 µg/ml ampicilline. Plasmid DNA was prepared using the Qiaprep Spin Miniprep kit (Qiagen) following the manufacturer's instructions and used as template in control PCR experiments.

#### Amplification of JC virus non-coding control region DNA

The “Fusion” primer concept was developed by Roche as part of the amplicon 454 sequencing protocol. The 5′ end of both forward and reverse “Fusion” primers is a 25-mer dictated by the requirements for the 454 sequencing system (primer A and primer B, respectively; see guidelines for amplicon experimental design, Roche) followed by the sequence key “TCAG” and a multiplex identifier sequence (MID) for sample identification. The 3′ portion of the primers consists of target-specific sequences. Here, we designed Fusion primers to amplify a ∼483 bp DNA fragment including the JC virus NCCR. The 3′end specific sequences were: 5′ gattcctccctattcagcactttg 3′ (Fwd primer) and 5′ tccactccaggttttactaa 3′ (Rev primer). All primers were synthesized at IDT Technologies (Belgium).

PCR was performed on both control plasmid DNA and on viral DNA from urine. All PCRs were run in triplicate and triplicates were pooled afterwards. For each sample a PCR pre-mix was prepared: 10 µl of Phusion high fidelity master mix (2×) (New England Biolabs), 1 µl forward Fusion primer (10 µM), 1 µl reverse Fusion primer (10 µM) and 6 µl of PCR-grade water. 2 µl of plasmid DNA preparation (*i.e.* ∼100 000 calculated plasmid copies) or 2 µl of extracted viral DNA was used as template (*i.e.* 20 µl final PCR volume). PCR was run under the following cycling conditions: 98°C for 30 seconds followed by 40 cycles of 98°C for 10sec, 60°C for 20 sec and 72°C for 20 sec and a final step at 72°C for 5 min. DNA amplicons were analyzed by agarose gel electrophoresis (pre stained 1.2% e-gel, Invitrogen), purified with AMPure XP beads (Agencourt) according to the manufacturer′s guidelines and eluted in low (10%) TE buffer. DNA was quantified using the Quant-iT picogreen assay kit (Invitrogen). Integrity of the DNA was confirmed on the Bioanalyzer 2100 (Agilent).

#### Basic amplicon sequencing on Roche GS-Junior platform and data analysis

Equimolar amounts of purified JC virus NCCR amplicons were pooled and sequenced on the GS Junior platform. 454 sequencing was performed at VIB Nucleomics core [http://www.nucleomics.be/, Leuven, Belgium]. All retrieved DNA sequences were subject to quality assessment. DNA sequences with high quality and covering the full JC virus NCCR were mapped onto the reference sequence (*i.e.* NCCR from CY isolate, NCBI acc.nr. AB038249). Raw sequence data from the 454 sequencing experiments have been submitted to the SRA database (accession number SRP023493).

#### 454 sequencing: assay performance and criteria set

To demonstrate the sensitivity and reliability of our 454 sequencing approach several control samples were included in the study design. Plasmid DNA carrying the archetype JC virus NCCR (NCBI AB038249) was used as template for PCR (∼100 000 calculated input copies) which allowed to evaluate for possible errors that can be introduced during the amplification step as well as 454 sequencing errors. To minimize this error rate all PCRs were performed in triplicate, and triplicates were pooled afterwards. Sensitivity of the assay was examined by spiking in a similar plasmid DNA, but harboring a predefined 66 base pair deletion at different ratios (*i.e.* 10%, 3%, 1% and 0.3%, respectively) before PCR. A considerable number of non-genuine DNA variations (*i.e.* deletions, insertions and single nucleotide variations) were present in less than 1% of the analyzed sequences retrieved for the control samples (data not shown) which is generally considered as background variation in 454 sequencing. To clean up our data and to increase reliability of the DNA variations observed, three criteria were set to evaluate potential DNA variations: 1) the background error rate was set at 1% meaning that only DNA variations detected in at least 1% of the viral sequences within a given sample are kept, 2) such variations should be detected by both forward and reverse sequenced DNA molecules at 1% and 3) DNA variations at homopolymeric DNA motifs cannot be reliably interpreted. The control samples in which the predefined 66 bp deletion was spiked in before PCR could be accurately detected down to the level of ∼1% while no other false deletions and/or single nucleotide variations were observed. From the control experiments it was concluded that our approach was reliable.

### Nested PCR for validation of deletions in JC virus NCCR DNA

Viral DNA was extracted from urine samples of HS6 and HS11 as described above. Of note, HS11 was one of the healthy subjects that donated several consecutive urine samples within a time interval of ∼1 year [at time point T0 (base line), T1 (∼7 months later), T2 (∼8 months later) and T3 (∼9.5 months later)]. First, outer PCR was performed on viral DNA using the same primers as for Sanger sequencing. A PCR pre-mix was prepared: 10 µl of Phusion high fidelity master mix (2×) (New England Biolabs), 1 µl forward primer (10 µM), 1 µl reverse primer (10 µM) and 3 µl of PCR-grade water. 5 µl of viral DNA was added as template and PCR was run: 98°C for 30 seconds followed by 40 cycles of 98°C for 10 sec, 60°C for 20 sec and 72°C for 20 sec and a final step at 72°C for 5 min. The outer PCR product was diluted 1∶100 in distilled water and used as template for two separate inner PCRs: one using the primers Fwdb (5′ aaggtagggaggagctg 3′) and Revb (5′ cccttgtgctaggggtt 3′) and one using the primers Fwdb and Rev2b (5′ cccttgtgctttgtttactt 3′). Inner PCRs were run as follows: 98°C for 30 seconds followed by 30 cycles of 98°C for 10 sec, 60°C for 15 sec and 72°C for 15 sec and a final step at 72°C for 5 min. DNA amplicons were analyzed on pre-stained agarose gels (4% e-gel, Invitrogen).

One µl of viral DNA from urine of HS53 was used in an outer PCR as described above. 1∶100 diluted PCR product was used as template for two inner PCRs with primers Fwdb (5′ aaggtagggaggagctg 3′) and Rev (5′ tatgggaggggtttcact 3′) or with primers Fwdb and Rev (5′ tatgggaggggcagtg 3′), respectively. Cycling conditions were: 98°C for 30 seconds followed by 30 cycles of 98°C for 10 sec, 70°C for 15 sec and 72°C for 15 sec and a final step at 72°C for 5 min. DNA amplicons were analyzed on pre-stained agarose gels (4% e-gel, Invitrogen).

## Results

### Sanger sequencing of the JC virus NCCR and VP1 coding sequence

**Figure 1:**
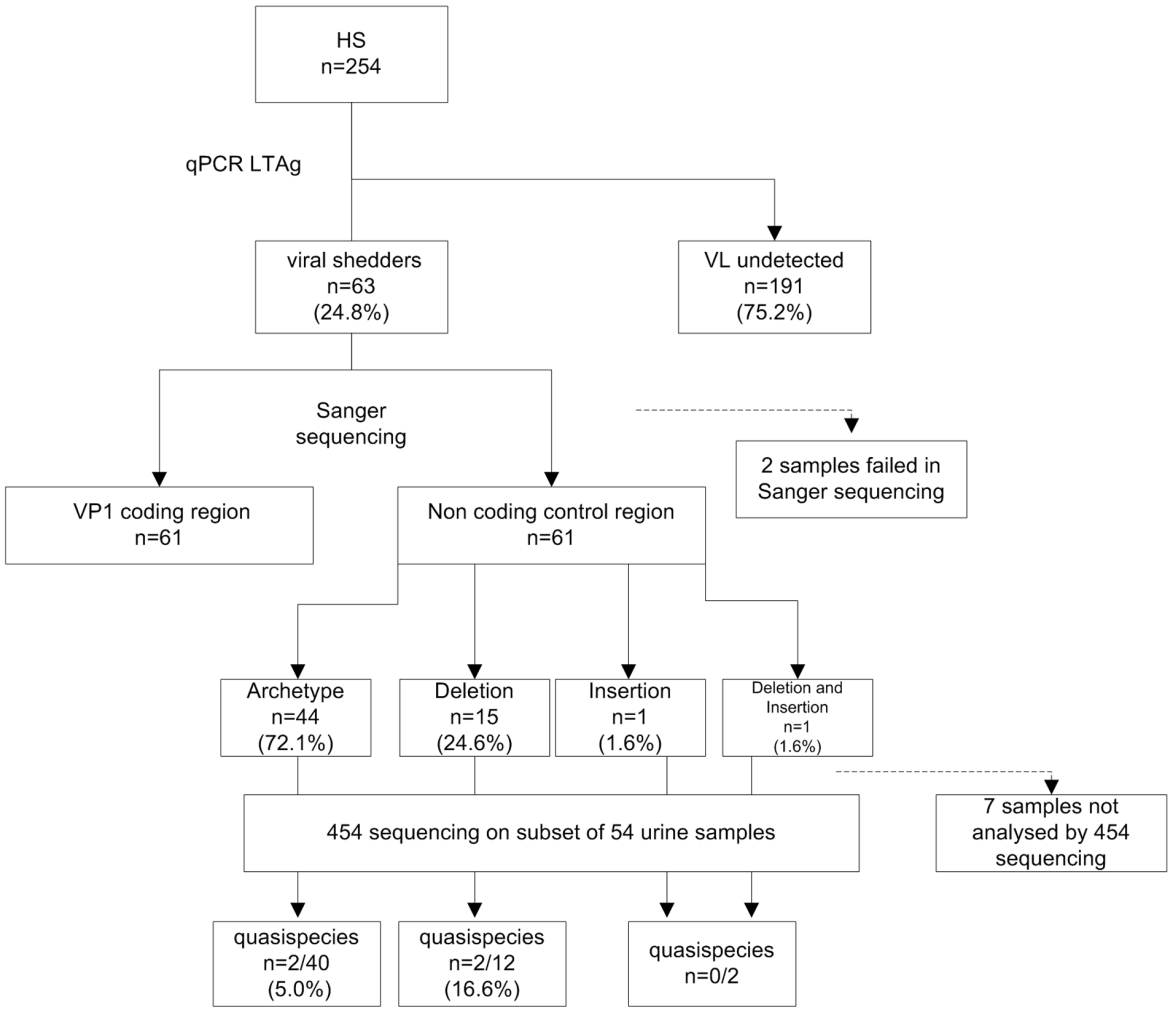
Outline of the experimental work presented. JC virus DNA was detected in urine of 63 out of 254 healthy subjects (HSs) by qPCR targeting the large T antigen (LTAg) gene. For 61 viral shedders the full length VP1 coding sequence and the non-coding control region (NCCR) DNA was obtained via Sanger sequencing. When compared to a reference archetype NCCR (*i.e.* CY isolate, NCBI acc.nr. AB038249) 44 samples did not contain any rearrangement while 15 samples (24.6%) harbored a deletion (between 1 and 28 bp), 1 sample (1.6%) carried a duplication in the consensus NCCR sequence and 1 sample carried both an insert and a deletion. A subset of 54 urine JCV DNA samples were further analyzed by next generation sequencing (454 pyrosequencing) to specifically look at the presence of JCV quasispecies that were identified in 2 out of 40 archetype samples (5.0%) and 2 out of 12 samples (16.6%) that contained a deletion when analyzed by Sanger sequencing. No quasispecies were detected in both samples carrying a duplication in the consensus NCCR.

An overview of the experimental set up is given in Figure 1. Urine samples were donated by 254 HSs. All samples were screened for the presence of JC virus DNA by quantitative PCR. 63 out of 254 HSs (∼24.8%) had shed viral DNA into their urine. A median of 6.60 log copies/ml (range 2.18–7.93) viral load (VL) was determined for these samples. Two µl of extracted viral DNA was used as template for Sanger sequencing of the NCCR. Detailed NCCR sequence information for those samples deviating from the archetype sequence by means of deletions and/or insertions is available in Figure S1. Two samples failed during the procedure likely due to their relatively low VL (2.18 and 2.27 log copies/ml, respectively). In 15 out of 61 samples (∼24.6%) small deletions between 1 and 28 bp were identified in the NCCR while insertions (duplications of 8 and 14 bp, respectively) were present in only two samples (3.3%). One of these duplications was assigned to a sample that also harbored a deletion in the NCCR (HS33, Table 1). Relatively few single nucleotide changes were identified in the NCCR. Only at 6 different positions a single nucleotide variation was present compared to the Japanese CY isolate archetype reference and this variation was mostly restricted to one or two samples. One exception was at nucleotide position 217 where most samples deviated from the reference sequence (217-A instead of 217-G). This nucleotide change has previously been shown to be a common feature of European and Asian JCV strains [23], [27].

**Table 1:** Overview of JC virus NCCR rearrangements (*i.e.* deletions and insertions) identified by Sanger sequencing and 454 sequencing.

NCCR consensus					Quasispecies	
*Sanger sequencing*					*454 sequencing*	
(n = 61)					(n = 54)	
			sample	rearrangement	sample	minority species
1. archetype			All, except under 2, 3 and 4	none	HS26	del 160 (∼2%;d)
(n = 44)					HS53	del 142–156 (∼6,5%; d)
2. deletions			HS11, HS37, HS40,HS55,HS61	del 165 (d)	HS11	del 162–189 (∼1,5%; d–e)
(n = 15)			HS20	del 126–153 (d)		
			HS31	del 106–116 (c–d)		
			HS7, HS9	del 218–222 (f)		
			HS5, HS23, HS43, HS51, HS57	del 221–222 (f)		
			HS45	del 216–243 (f)	HS45	archetype (∼5%)
3. insertions			HS46	ins 216–14bp (f)		
(n = 1)						
4. insertion + deletion			HS33	ins 74–8bp (c) + del 221–222 (f)		none
(n = 1)						

Comparison of the consensus NCCR as determined by Sanger sequencing (n = 61) revealed four distinct groups of samples: 1) archetype sequences with no DNA rearrangements compared to the reference NCCR (CY isolate), 2) samples harboring polymorphic deletions, 3) samples carrying polymorphic insertions and 4) sample harboring both a deletion and an insertion. 454 sequencing was further applied (n = 54) to identify JCV quasispecies. Healthy subject (HS) samples in which quasispecies were identified, the nature of the minority sequences contributing to the quasispecies and the percentage of viral NCCR sequences harboring the minority sequences are indicated. The sequence blocks (a to f) affected by the NCCR rearrangements are indicated between brackets. Of note, HS11 carries a 1 bp deletion at position 165 that in ∼1.5% of the viral population is part of a more extensive deletion. The numbering scheme is based on [12].

**Figure 2:**
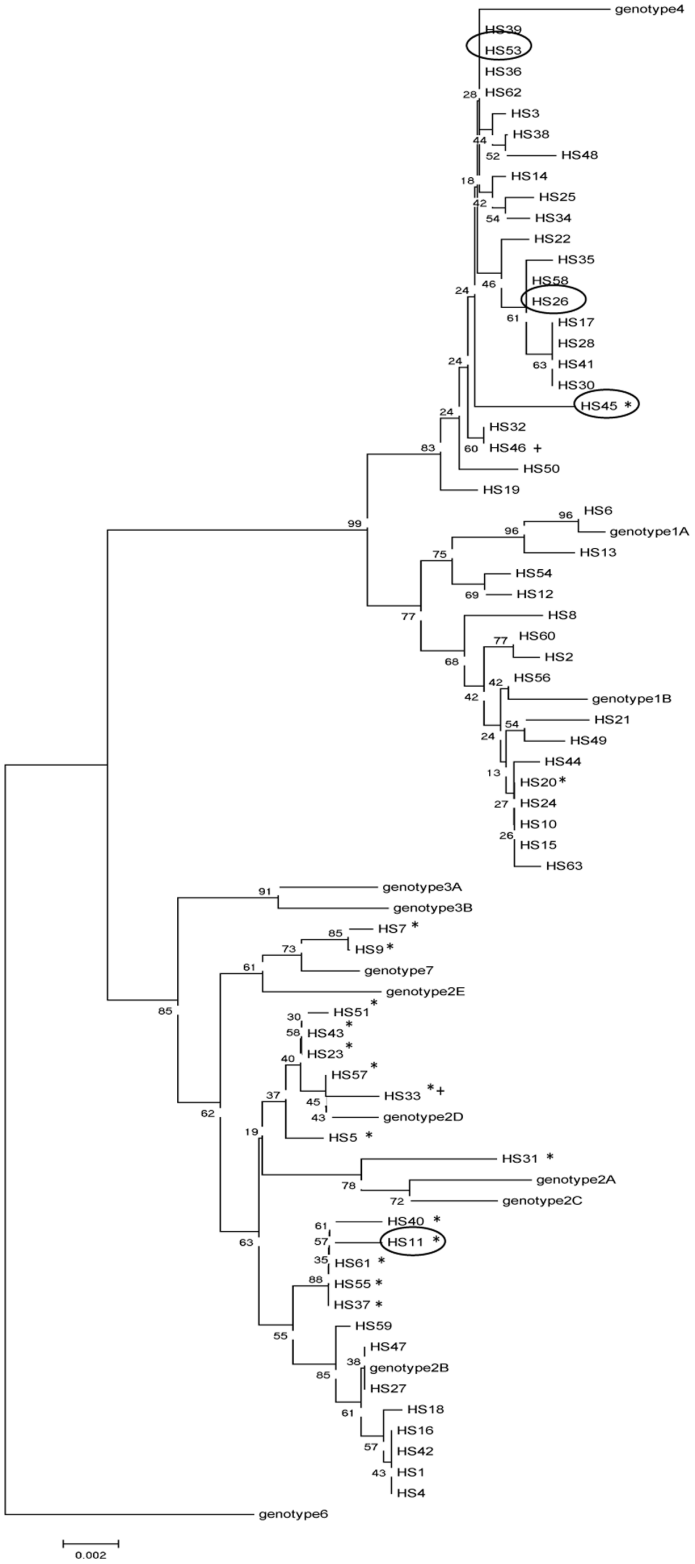
Phylogenetic analysis based on the VP1 coding sequence from healthy subjects. The full length VP1 coding sequence (1065 bp) was obtained for all healthy subjects for which also the non-coding control region (NCCR) was Sanger sequenced (n = 61). The relatedness of the HS samples to defined JC virus genotypes is illustrated in a phylogenetic tree. For each JCV genotype the following reference VP1 coding sequences were used (NCBI accession number between brackets; reference sequences taken from [26]): genotype 1A (AF015526), genotype 1B (AF015527), genotype 2A (AF015529), genotype 2B (AF015533), genotype 2C (AF015535), genotype 2D (AF015536), genotype 2E (AF281606), genotype 3A (U73500), genotype 3B (U73501), genotype 4 (AF015528), genotype 6 (AF015537), genotype 7 (U61771). Healthy subjects (HS) in which deletions or insertions were identified in the non-coding control region via Sanger sequencing are indicated by * (deletion) or + (insertion). HSs in which JCV quasispecies were identified by 454 sequencing are circled.

Based on the obtained full length VP1 coding sequences (1065bp) phylogenetic analysis was performed to assign a JCV genotype to the different samples (Figure 2). To build this tree reference genotype sequences were used as described in [26]. Most of the samples were divided over genotype 1, 2 and 4 while 2 samples clustered together with genotype 7 and no obvious genotype 3 nor genotype 6 samples were present. Eleven out of the fifteen samples carrying a polymorphic deletion in the consensus NCCR were related to genotype 2 JCV while 2 of those samples were genotype 7. The remaining samples were genotype 1 and 4, respectively. Both samples harboring a duplication belonged to genotypes 2 and 4 (Figure 2).

### Identification of JC virus NCCR quasispecies in urine of healthy subjects

For a total of 54 urine JCV DNA samples an interpretable 454 amplicon sequencing was obtained. Two µl of viral DNA (with variable viral load) was used as template for PCR (performed in triplicate) which implies that at least ∼4789 input copies were used for 454 sequencing (Min: 4789; Max: 19,060,878; Mean: 3,013,724; and Median: 1,358,573) (Table S1). A total of 187,420 quality checked DNA sequences covering the full length JCV NCCR were retrieved and divided over 59 samples (*i.e.* 54 urine samples and 5 plasmid control samples, see Materials and Methods) resulting in an average coverage depth between 1,457 (min.) and 7,044× (max.) per nucleotide position. Due to the relatively high viral load for most of the urine samples, no oversampling (*i.e.* sequencing the same DNA molecule multiple times) occurred. Hence, no sequencing bias was introduced in our data set (Table S1).

For all samples run in the 454 sequencing protocol obtained full length NCCR DNA sequences were mapped against the archetype reference sequence. The number of reads retrieved for each sample is given in Table S1. This approach allowed deducing the consensus NCCR sequence for all analyzed samples, which represents the most abundant viral species (NCCR sequence) present in a given sample. Importantly, all polymorphic deletions and insertions as they were identified by Sanger sequencing were recovered in these consensus sequences. Beyond the identification of the NCCR consensus sequence, our main goal was to investigate the presence of JCV quasispecies *i.e.* the identification of additional viral variants within a single sample present at a lower frequency than the consensus NCCR sequence (further referred to as minority viral species). Therefore, for each sample all retrieved NCCR sequences deviating from the reference sequence were analyzed under the criteria set for sensitivity and reliability of our assay (see Materials and Methods). Hence, only DNA variations present in at least 1% of the total number of assigned sequences and supported by both forward and reverse sequencing were considered genuine variations. Overall, after analysis of the 454 sequencing data 4 samples out of 54 (7.4%) were identified as containing quasispecies (Figure 1 and Table 1). Two out of 40 HS without any rearrangements in the consensus NCCR carried deletions present in 2% (HS26, 46 y old women) and 6.5% (HS53, 48 y old women) of the viral sequences, respectively. Both HS had a high JCV VL of 7.3 and 6.1 log copies/ml urine, respectively. In addition, 2 out of 12 HS harboring a deletion in the consensus NCCR contained quasispecies (HS11; a 53 y old man with a VL of 6.6 log copies/ml and HS45; a 60 y old women with a VL of 7.2 log copies/ml). Of note, for HS11 multiple consecutive urine samples were collected in which persistent viral shedding was found (see also below). No NCCR quasispecies were identified in both samples carrying a DNA duplication in the consensus non-coding control region (Table 1; HS46 and HS33). Three out of four samples with assigned quasispecies harbored a deletion present in only a minority of sequences. In one of these samples the 1 bp deletion identified by Sanger sequencing (HS11, del 165, Table 1) was part of the larger deletion (del162–189) identified as minor virus variant in this sample. Of interest, sample HS45 was peculiar since a relatively large 28 bp deletion was identified in 95% of all analyzed sequences after mapping against the reference sequence. Hence, still ∼5% of the NCCR sequences obtained for this sample perfectly matched when compared with the reference archetype sequence.

Next to DNA rearrangements, the intra-sample single nucleotide variability was also investigated by 454 sequencing in search for potential quasispecies characterized by nucleotide replacements. When applying our stringency settings to evaluate the presence of genuine DNA variations single nucleotide replacements could not be identified in any of the samples. Hence, it appeared that no minority viral variants characterized by specific nucleotide replacements were present in the 54 urine samples analyzed by next generation sequencing.

### Validation of JC virus quasispecies identification in urine of healthy subjects

**Figure 3:**
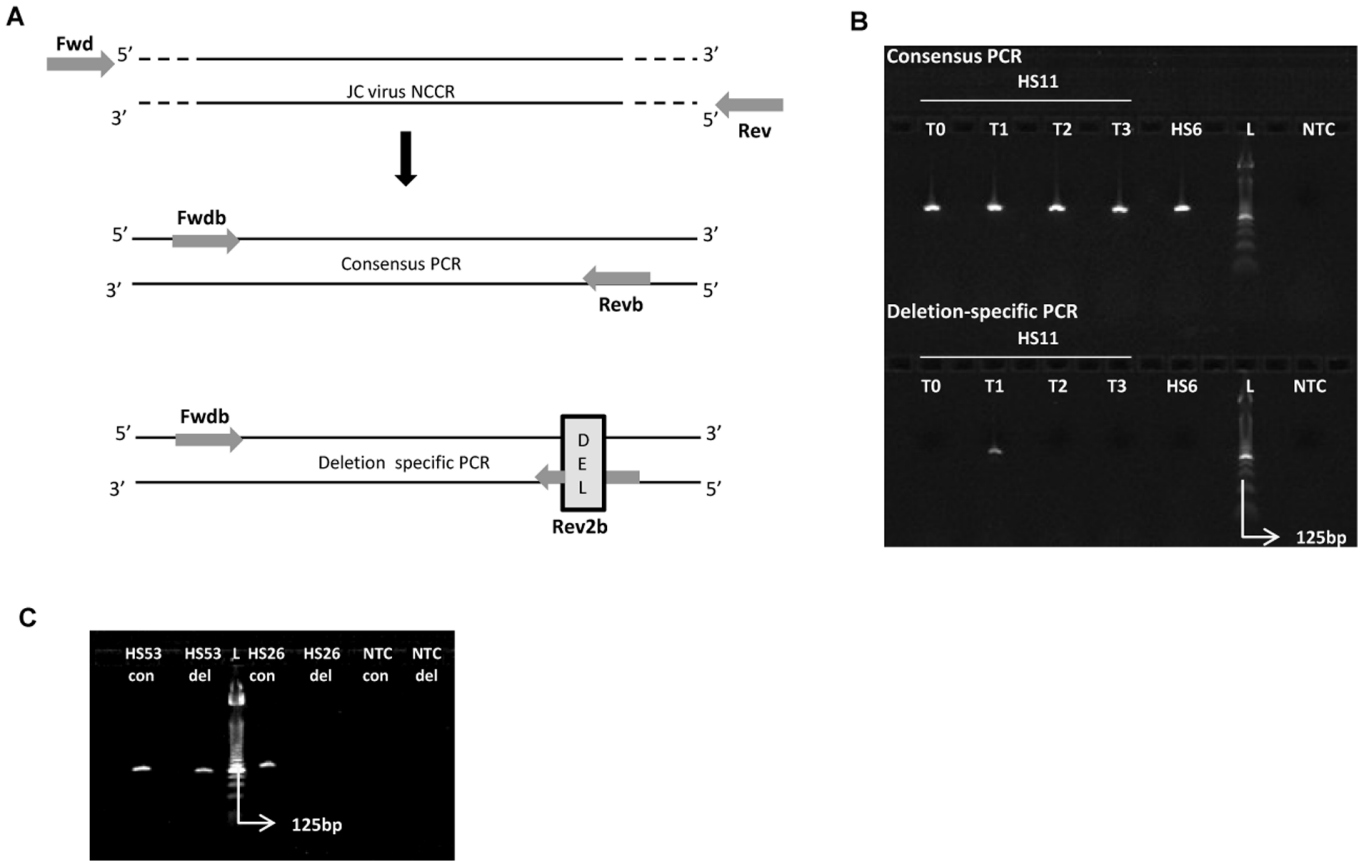
Validation of JC virus quasispecies by nested PCR. (A) A nested PCR approach was developed to validate the presence of the 28 bps deletion identified in ∼1.5% of the viral DNA sequences in HS11. (B) The nested PCR approach was applied on viral DNA extracted from urine from HS11. Urine aliquots donated at different time points were included: T0, T1, T2 and T3. Viral DNA extracted from HS6 T1 was included as a negative control, together with a no template control (NTC). The upper panel shows successful amplification of the consensus DNA fragment in all samples. In contrast, only in HS11 T1 a PCR fragment could be amplified when using the reverse primer spanning the deletion instead of the consensus reverse primer, confirming the presence of this deletion. L = 25 base pair DNA ladder (Invitrogen). (C) A similar nested PCR approach was used to demonstrate the existence of quasispecies in HS53. DNA fragments were generated when a primer set designed to amplify a consensus sequence from both HS53 and HS26 (lanes HS53 con and HS26 con) were used. When the reverse primer was replaced by a primer specifically targeting the sequence deleted in the quasispecies succesfull amplification was only detected in HS53 (HS53 del), but not in HS26 (HS26 del). NTC: no template control. L = 25 base pair DNA ladder (Invitrogen).

In order to validate the presence of JC virus quasispecies in HS11 (*i.e.* a 28 bp deletion present in ∼1.5% of the viral DNA sequences, Table 1) a nested PCR approach was followed. Viral DNA was freshly prepared from the following urine samples: HS11 T0 (viral load 7.3 log copies/ml), HS11 T1 (*i.e.* the urine collection also used for 454 sequencing; viral load 6.6 log copies/ml), HS11 T2 (viral load 7.8 log copies/ml) and HS11 T3 (viral load 7.7 log copies/ml) which gave the opportunity to follow the presence of the detected deletion over time. A nested PCR approach was applied on these samples as described under Methods and graphically explained in Figure 3A. As a negative control, viral DNA extracted from HS6 and in which the 28 bps deletion was not detected by 454 sequencing, was included in the assay. As expected a JC virus NCCR DNA fragment was amplified during inner PCR when primers designed to target a consensus fragment, present in both HS6 and HS11, were used (Figure 3B; upper panel). However, only in sample HS11 T1 a DNA fragment was successfully amplified during inner PCR in which the consensus reverse primer was replaced with a primer over-spanning the potential deletion (Figure 3B; lower panel). This result confirms that the quasispecies detected in HS11 truly exists and can be specifically attributed to this sample, but in this case is likely to be a transient phenomenon as it was not detected in later samples. Using a similar nested PCR approach the 15 bp deletion identified in ∼6.5% of the DNA sequences from HS53 (Table 1) were also confirmed to be genuine and specific for HS53 (Figure 3C).

## Discussion

The non-coding control region interspersed between early and late coding genes on the JC virus genome is known to play a pivotal role in viral replication by regulating early gene expression and by being susceptible to host transcription factors [15], [17], [24]. DNA rearrangements within this region are strongly linked with PML indicating that the NCCR might be a viral determinant supporting viral growth in infected glial cells. In line with this view, several reports have demonstrated increased *in vitro* early gene transcription and viral replication driven by naturally occurring rearranged JCV NCCR sequences taken from PML-cases [17], [22]. Whether such rearrangements emerge and out-compete archetype NCCR during PML progression or, alternatively, if PML is caused by infection of glial cells with pre-existing rearranged JC virus, are still a matter of debate. We reasoned that when rearrangements in the NCCR would occur within an infected host (*i.e.* if JCV quasispecies would exist) this might add to the risk of changing viral tropism, for instance due to an altered sensitivity of the NCCR for cell-specific transcription factors. Therefore, the rationale of this study was to examine to what extent variation in the NCCR and NCCR quasispecies occur within healthy subjects. For this, HSs were selected based on the presence of viral DNA in their urine. Since viral DNA present in urine is likely to be a transmittable form of JCV [18], [28] studying NCCR rearrangements seems also of particular interest here.

Our scope was not limited to study the variation observed in the consensus NCCR sequences, which has been addressed previously in different cohorts by Sanger sequencing [23], [29], [30], but was mainly focused on investigating quasispecies present in the host by applying next generation sequencing. Due to its ability to generate relatively long DNA sequences, a crucial feature to reliably detect insertions and deletions within a DNA sequence, 454 (pyro)-sequencing was the platform of choice. Stringent criteria were set for analysis purposes to demonstrate the sensitivity and reliability of the assay allowing the detection of NCCR variability even if it was present in only a minor fraction of the sequenced viral population. No common genomic alterations were identified in the minority viral species contributing to the quasispecies (Table 1). However, a main feature of most of the genomic alterations, both defining the consensus NCCR as well as the minority viral species, was the deletion of part of the NCCR. Our data seem to be in line with a current notion that deletions precede duplication events during the NCCR rearrangement process [31] as has been previously suggested for PML-associated JCV [21].

Our observations might shed some light on the molecular evolution of JC virus within a naturally infected host. It has been suggested previously that during the course of JC virus evolution sporadic genomic rearrangements in the NCCR occur and might ultimately become dominant over the archetype in some JCV lineages or sub-lineages [23]. The presence of a unique, sporadic JCV NCCR rearrangement characterized by a 36 nucleotide insertion unrelated to the genomic alterations presented in this manuscript, and previously reported to be present in the urine of a healthy donor [30] can be readily explained in a similar way. The polymorphic deletions and insertions identified in ∼28% of the analyzed samples (Figure 1) in our sample set seem to further support this idea. If assumed that the archetype NCCR represents an ancient form of JC virus from which all rearranged forms are derived [19], [28], any deletion and/or insertion that goes along with more efficient viral replication (*e.g.* via enhancing transcription of LTAg) might become positively selected within a specific cellular context, likely due to the loss of negative or the creation of positive transcriptional control signals [17], [22]. Although we cannot discriminate between scenarios in which the polymorphic rearrangements identified in HSs resulted from an intra-host natural selection or were the consequence of an infection with a pre-existing rearranged JCV strain, the observation made in one of our samples seems to be in favor of the first possibility. As shown, one of the HSs was characterized as a viral shedder in which ∼95% of the NCCR DNA copies shed into the urine carried a 28 base pairs deletion (Table 1), implying that ∼5% of the JC virus DNA still matched with the archetype reference sequence. Therefore, it seems likely that this specific rearrangement indeed has become positively selected and might potentially out-compete the non-rearranged archetype form. Similar observations were made in plasma from immune suppressed kidney transplant patients where rearranged BKV NCCR variants emerged and replaced the archetype BKV as majority (consensus) species *in vivo* during persistent viremia [32]. Assuming that JCV present in urine is an infectious and transmittable form [28], such sporadic rearrangement might have the potential to start spreading and circulating within a human population, providing a possible explanation for observations made by others [23], [29].

Another interesting observation came from HS11 in which a 28 base pairs deletion was identified in ∼1.5% of the viral population isolated from urine at a given time point (T1). By applying a nested PCR approach we showed that this particular variant was genuine, but cleared from the viral population (or at least became undetectable in our assay), by a yet unknown mechanism, about 1 month later (at time point T2 and in subsequent samples). It has been hypothesized before that most alterations in the JCV NCCR need to be categorized as “dead end” meaning that they are only transiently present in the viral population [18]. Mechanisms by why some NCCR rearrangements become positively selected while others are cleared from the population are yet to be fully understood. A concerted interplay of viral determinants, host cell factors and antiviral immune responses is likely to add to this selection. Evidently, if the NCCR deletion specified here for HS11 would cause suppressed viral replication compared to the archetype JCV it would be expected to ultimately disappear. Most NCCR rearrangements studied so far, both for JCV and BKV and including even relatively modest alterations (*i.e.* single deletions or duplications) were shown to significantly increase early gene transcription and replication in a variety of cell lines [17], [22], [32], [33]. It should be noted however, that most of these sequences originated from patient samples (*i.e.* JCV PML cases, BKV kidney transplant recipients) creating a bias towards positive selection of highly replicating rearranged viral strains. Hence, non-functional, negatively selected NCCR sequences are less likely to be identified, but nevertheless likely to exist. Even more, the relatively low number of JCV quasispecies identified by 454 sequencing might be an underestimation of the true susceptibility of the NCCR for genomic rearrangements due to the transient nature of most of the emerging viral quasispecies. Also the cellular environment in which virus replication takes place is likely to be an additional feature driving quasispecies emergence. Whereas JCV replication is dependent on host cell DNA polymerase and, hence, occurs at high fidelity in most permissive cell types, the DNA recombination apparatus present in B-lymphocytes might support rearrangement of the NCCR [31] potentially rendering them more prone to quasispecies detection.

Host antiviral immunity might also participate in controlling viral quasispecies [32]. An interesting concept was put forward stating that cellular immunity directed against early gene encoded epitopes may contribute in controlling the emergence of rearranged, highly replicating BKV in healthy immune competent individuals [32], possibly reflective of the relatively low number of quasispecies identified in our group of healthy subjects. Under conditions of immune suppression however, impaired T-cell activity might facilitate the emergence of rearranged BKV as shown for immune suppressed kidney transplant patients [32]. If such scenario holds true it merits further investigation to determine if intra-host born JCV NCCR rearrangements are more likely to happen in immune suppressed individuals compared to healthy subjects.

JC virus is the causative agent for PML. Although viral determinants contributing to the pathogenesis of PML remain poorly understood special interest has been paid to be the so-called domain d of the NCCR which turns out to frequently carry deletions in PML-associated samples [2], [17], [21]. In addition, *in vitro* cell assays also underline the importance of NCCR domain d in JC virus replication [17]. Of note, some of the deletions that we identified here are also located in or near domain d whereas the *ori*-proximal end of the NCCR turned out to be less prone to rearrangements. In addition to domain d also domain f was shown to be highly affected by deletion events (Table 1). Multiple studies have indicated that the JCV NCCR harbors various transcription factor binding sites, amongst which recognition sites for the NFI family of cellular DNA binding proteins, SP1, SPi-B, Tst-1/Oct-6/SCIP and AP-1 (c-jun) are located within domain d and f (see [18] and references therein for a comprehensive review on host transcription factors influencing JCV transcription). Clearly, rearrangements in this region might alter the binding capacity of the host transcription factors adding to changed viral tropism and altered gene transcription levels, a hypothesis that merits further investigation.

In conclusion, we investigated the presence of JC virus quasispecies in urine of healthy subjects and were able to demonstrate their existence in a minor part of the study population (∼7.4%). Taken together, from our work the idea emerges that the occurrence of DNA variability in the NCCR of JCV is a dynamic process with specific rearrangements becoming positively selected, while other variations are likely to be only transient. Whether these naturally occurring variants can alter the viral tropism and to what extent they might add to an increased risk for PML following opportunistic infection of the brain remains to be examined. Nevertheless our experimental approach turns out to be a valuable tool readily applicable for future investigations of JCV quasispecies in other cellular reservoirs or body fluids and under relevant clinical settings such as immune suppression.

## Supporting Information

Figure S1 **Sequence alignment of the non-coding control region DNA sequence retrieved by Sanger sequencing from viral DNA isolated from urine (n = 17).** Only samples in which DNA rearrangements (deletions or insertions) were identified in comparison to the archetype reference sequence are included in the alignment. DNA regions in which no rearrangements were present are not shown and are symbolized by//. On top of the alignment the archetype (AR) NCCR from CY isolate (267 nucleotides, NCBI acc.nr. AB038249) is presented. Deletions are indicated by *. Gaps (−) were introduced for proper alignment of the sequences. Single nucleotide changes (compared to the reference sequence) are shaded in grey. Nucleotide numbering of the NCCR is indicated on top of the alignment. The lower bar gives a schematic representation of the NCCR DNA architecture [18] showing in which predefined NCCR domain the identified rearrangements were present. *Ori*: origin of replication.
(TIF)

Table S1 **Viral load and input copy number for urine samples analyzed by 454 sequencing.** Viral DNA was extracted from urine samples from HSs and used as template for PCR and subsequent 454 sequencing as described under Methods. All samples were assigned a multiplex identifier (MID; see Materials and Methods) for analysis purposes. In total 187,420 DNA sequences (reads) covering the full length JCV NCCR were retrieved and mapped on the reference sequence (*i.e.* JCV NCCR archetype sequence, CY isolate, from NCBI accession number AB038249). The number of analyzed reads per urine sample is indicated. The JC virus viral load (JCV VL) as determined for the urine samples is expressed as log copies/ml. Based on this VL a sampling size *i.e.* number of analyzed reads divided by the input copy number was calculated.
(XLS)
