# Supplementary figures and images for: Quasispecies Analysis of JC Virus DNA Present in Urine of Healthy Subjects
Source: PLoS One. 2013 Aug 15;8(8):e70950. doi: 10.1371/journal.pone.0070950 (PMC3744523; doi:10.1371/journal.pone.0070950)

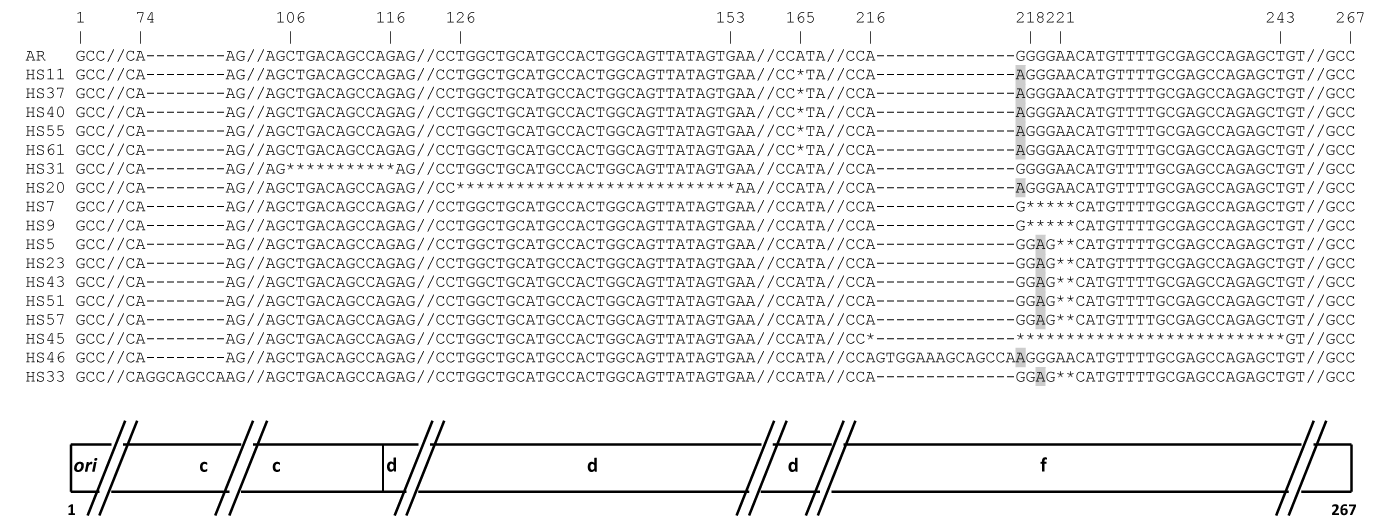

Supplement: Figure S1 — Sequence alignment of the non-coding control region DNA sequence retrieved by Sanger sequencing from viral DNA isolated from urine (n = 17). Only samples in which DNA rearrangements (deletions or insertions) were identified in comparison to the archetype reference sequence are included in the alignment. DNA regions in which no rearrangements were present are not shown and are symbolized by//. On top of the alignment the archetype (AR) NCCR from CY isolate (267 nucleotides, NCBI acc.nr. AB038249) is presented. Deletions are indicated by *. Gaps (−) were introduced for proper alignment of the sequences. Single nucleotide changes (compared to the reference sequence) are shaded in grey. Nucleotide numbering of the NCCR is indicated on top of the alignment. The lower bar gives a schematic representation of the NCCR DNA architecture [18] showing in which predefined NCCR domain the identified rearrangements were present. Ori: origin of replication. (TIF) [file pone.0070950.s001.tif]
